# Association of High and Low Molecular Weight Glutenin Subunits with Gluten Strength in Tetraploid Durum Wheat (*Triticum turgidum* spp. *Durum* L.)

**DOI:** 10.3390/plants12061416

**Published:** 2023-03-22

**Authors:** Jameel M. Al-Khayri, Rana M. Alshegaihi, ELsayed I. Mahgoub, Elsayed Mansour, Osama O. Atallah, Muhammad N. Sattar, Muneera Q. Al-Mssallem, Fatima M. Alessa, Mohammed I. Aldaej, Abdallah A. Hassanin

**Affiliations:** 1 Department of Agricultural Biotechnology, College of Agriculture and Food Sciences, King Faisal University, Al-Ahsa 31982, Saudi Arabia; maldaej@kfu.edu.sa; 2 Department of Biology, College of Science, University of Jeddah, Jeddah 21493, Saudi Arabia; 3 Genetics Department, Faculty of Agriculture, Zagazig University, Zagazig 44511, Egypt; 4 Department of Crop Science, Faculty of Agriculture, Zagazig University, Zagazig 44511, Egypt; sayed_mansour_84@yahoo.es; 5 Department of Plant Pathology, Faculty of Agriculture, Zagazig University, Zagazig 44511, Egypt; osamaoatall1h@ufl.edu; 6 Department of Plant Pathology & Microbiology, Faculty of Agriculture & Life Sciences, Texas A&M University, College Station, TX 77840, USA; 7 Central Laboratories, King Faisal University, Al-Ahsa 31982, Saudi Arabia; mnsattar@kfu.edu.sa (M.N.S.); mmssallem@kfu.edu.sa (M.Q.A.-M.); 8 Department of Food Science and Nutrition, College of Agriculture and Food Sciences, King Faisal University, Al-Ahsa 31982, Saudi Arabia; falissa@kfu.edu.sa

**Keywords:** durum wheat, dough strength, glutenin subunits, gluten strength, HMWGS, LMWGS

## Abstract

The gluten strength and the composition of high- and low-molecular-weight glutenin subunits (HMWGSs and LMWGSs) of fifty-one durum wheat genotypes were evaluated using sodium dodecyl sulfate (SDS) sedimentation testing and SDS polyacrylamide gel electrophoresis (SDS-PAGE). This study examined the allelic variability and the composition of HMWGSs and LMWGSs in *T. durum* wheat genotypes. SDS-PAGE was proven to be a successful method for identifying HMWGS and LMWGS alleles and their importance in determining the dough quality. The evaluated durum wheat genotypes with HMWGS alleles 7+8, 7+9, 13+16, and 17+18 were highly correlated with improved dough strength. The genotypes containing the LMW-2 allele displayed stronger gluten than those with the LMW-1 allele. The comparative in silico analysis indicated that Glu-A1, Glu-B1, and Glu-B3 possessed a typical primary structure. The study also revealed that the lower content of glutamine, proline, glycine, and tyrosineand the higher content of serine and valine in the Glu-A1 and Glu-B1 glutenin subunits, and the higher cysteine residues in Glu-B1 and lower arginine, isoleucine, and leucine in the Glu-B3 glutenin, are associated with the suitability of durum wheat for pasta making and the suitability of bread wheat with good bread-making quality. The phylogeny analysis reported that both Glu-B1 and Glu-B3 had a closer evolutionary relationship in bread and durum wheat, while the Glu-A1 was highly distinct. The results of the current research may help breeders to manage the quality of durum wheat genotypes by exploiting the allelic variation in glutenin. Computational analysis showed the presence of higher proportions of glutamine, glycine, proline, serine, and tyrosine than the other residues in both HMWGSs and LMWGSs. Thus, durum wheat genotype selection according to the presence of a few protein components effectively distinguishes the strongest from the weakest types of gluten.

## 1. Introduction

Durum wheat (*Triticum durum* Desaf.) (2n = 4x = 28) is one of the earliest domesticated plants worldwide. It was introduced in the Mediterranean Basin ~2000 years ago as a staple food and has been utilized in making various food products, including bread and pasta [1]. Variations in gluten and dough characteristics of wheat, especially the strength and extensibility, modulate its suitability for various end-uses [2]. The specific composition and suitable quantity of gluten-forming proteins in wheat grains modulate the changes in dough properties. Wheat grains contain 8 to 20% protein, of which gluten proteins represent about 85% and are responsible for flour elasticity [3,4]. Wheat grains are a rich source of gluten proteins, divided into two major groups, i.e., glutenins and gliadins. Glutenins and gliadins constitute the gluten polymeric and monomeric fractions and are responsible for dough elasticity and extensibility, respectively [5]. Glutenin subunits (GS) are further divided into high molecular weight glutenin subunits (HMWGSs) and low molecular weight glutenin subunits (LMWGSs) according to their mobility in sodium dodecyl sulfate-polyacrylamide gel electrophoresis (SDS-PAGE) [6].

The genes located at Glu-A1,Glu-B1, and Glu-D1 loci on the long arms of the homologous chromosomes 1A, 1B, and 1D encode one or more protein subunits of HMWGSs [7,8]. Compared to HMWGSs, the LMWGSs are controlled by multigenes and are more abundant and complex. A multigene family located on the short arms of chromosomes 1A, 1B, and 1D at the Glu-A3, Glu-B2, Glu-B3, and Glu-D3loci encode LMWGS proteins [9]. Moreover, LMWGSs are strongly linked with γ and ω-gliadins at the Gli-1 locus [10]. Higher gluten strength is essential in pasta products as it ensures good cooking quality. Several methods can be utilized to assess the gluten strength, such as the mixograph, farinograph, and SDS-sedimentation test [10,11,12]. In addition, gliadin proteins are essential to predict the strength of the dough based on gluten polypeptides (gliadins and glutenins) in durum wheat. Gliadin 42, for example, is associated with weak gluten, while gliadin 45 correlates with strong gluten [13]. These gliadin bands are coded by alleles located on the short arm of chromosome 1B at the Gli-B1 locus and genetically correlated with groups of LMWGSs encoded by genes at the Glu-B3 locus [14].

It has been reported that LMWGSs are functionally involved in the viscoelasticity of gluten in durum wheat [15,16,17]. Contrarily, some other studies explored the relationship between the quality parameters and the existence of specific HMWGSs in durum wheat. This may be in part due to the presence of the null allele at the Glu-A1 locus in most durum wheat genotypes. Attempts to examine the relationship between the viscoelasticity of durum wheat gluten and HMWGSs showed no relationship between the HMWGS and the gluten properties [18]. Due to innovative molecular biology tools and protein separation techniques, research studies have shifted from wheat cultivar assessment for phenotypic traits and glutenin content to developing new cultivars with specific glutenin combinations [19,20]. Developing cultivars with vital elastic gluten is critical in durum wheat breeding. Future breeding of wheat varieties with improved bread-making quality will be greatly facilitated by an understanding of the genetic basis of endosperm storage proteins, which are largely responsible for varietal differences in viscoelasticity. This study determined the electrophoretic variability of durum wheat endosperm proteins and the linkage relationships between the genes encoding them to evaluate the possible use of the electrophoretic banding patterns of HMW glutenin subunits for predicting baking quality in the breeding program.Thus, this research was conducted to (i) evaluate the association of the Glu-A1, Glu-B1, and Glu- B3 loci with the gluten properties in tetraploid durum wheat genotypes, (ii) predict the efficacy of selected HMWGS and LMWGS genes in enhancing gluten quality, and (iii) compare the amino acid composition and sequences of Glu-A1, Glu-B1, and Glu-B3 in both bread and durum wheat genotypes using phylogenetic and computational analyses. This will shed light onto the amino acids and endosperm protein properties that should be included in potential hybrids of future breeding programs.

## 2. Results

### 2.1. Variability of HMWGS and LMWGS

**Figure 1:**
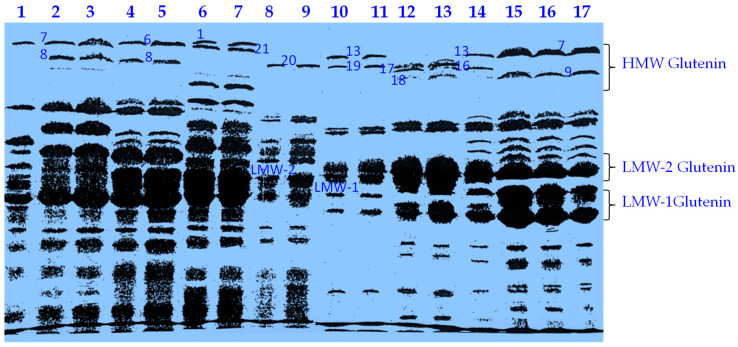
A sample SDS-PAGE output with some HMWGSs and LMWGSs found in studied tetraploid durum wheat genotypes. Lanes from 1 to 17 are genotypes 1, 3, 4, 7, 10, 13, 18, 22, 23, 27, 29, 32, 36, 42, 47, 49, and 51, respectively.

Each evaluated durum wheat genotype contained one LMWGS. The number of HMWGSs (Glu-A1 and Glu-B1) ranged from 1 to 3 per genotype, in addition to 1 Glu-B3glutenin subunit. The relationship of alleles at all pairs of loci was not significant according to chi-square values (*p* > 0.05), suggesting that most linkage disequilibria may be eliminated by random mating. The evaluated genotypes were classified into 17 groups based on HMWGSs and LMWGSs. The seventeen different HMWGS and LMWGS classes observed in the studied durum wheat genotypes are presented in Table 1. The genotypic classification was based on Glu-A1, Glu-B1, and Glu-B3 loci alleles. These involved 2Glu-A1 (null and 1), 10 Glu-B1 (7, 7+8, 7+9, 6+8, 13+16, 20, 13+19, 14+15, 17+18, and 21), and 2Glu-B3 (LMW-1 and LMW-2) allelic variants (Figure 1). The most common combination in 7 durum wheat genotypes was null, 7, and LMW-1.

**Figure 2:**
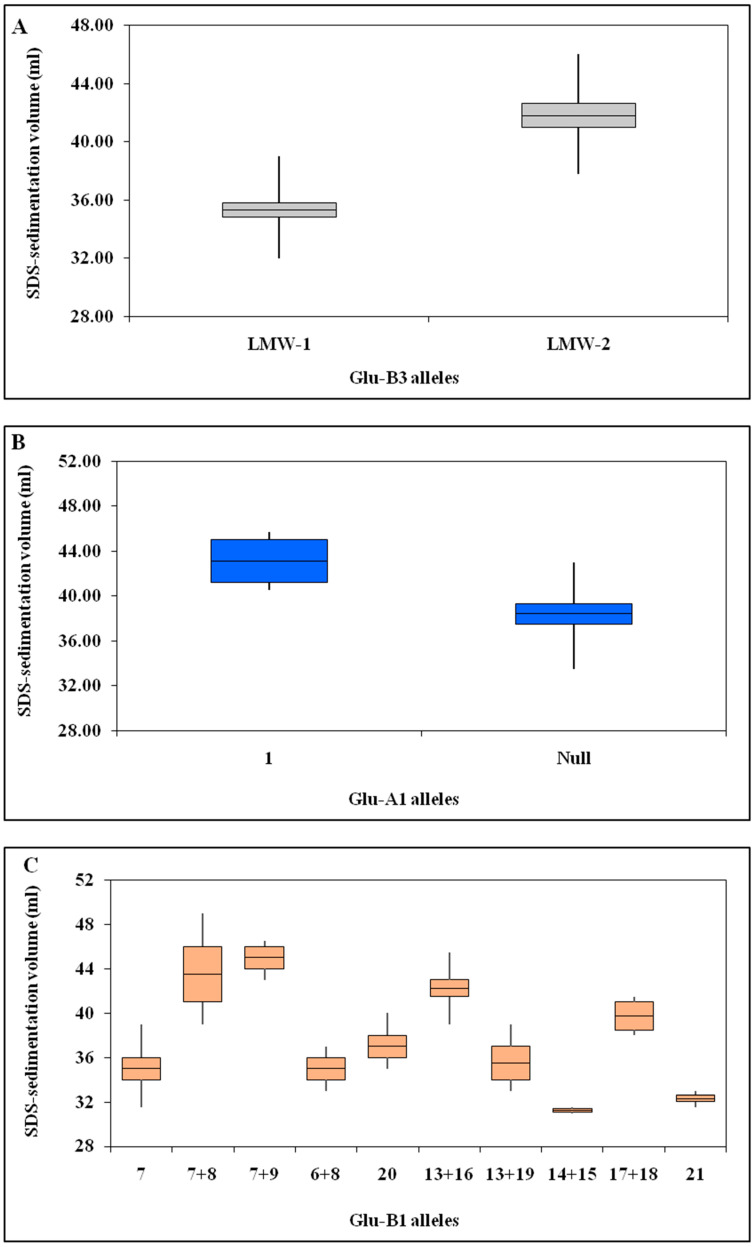
Effects of allelic variation on gluten strength at the Glu-B3 locus(**A**) Glu-A1 locus (**B**), and Glu-B1 locus (**C**).

Mean protein content and SDS sedimentation volumes of the analyzed wheat are grouped according to allelic composition at the tested HMWGS and LMWGS loci (Table 2). The existence of particular HMWGS and LMWGS alleles at the three loci as measured by the SDS sedimentation test are also presented in Figure 2. Mean SDS sedimentation volumes of genotypes possessing the LMW-2 alleles at the Glu-B3 locus were significantly higher than those containing the LMW-1 allele (Figure 2A). In addition, the HMWGS of the Glu-A1 locus was correlated with a higher sedimentation volume than the null allele (Figure 2B). Comparison studies of the quality effects of allelic variants at the locus of Glu-B1proved that genotypes possessing the HMWGSs 7+8, 7+9, 17+18, or 13+16 had higher SDS sedimentation volumes than those containing the subunits 7, 6+8, 20, 14+15, 13+19 or 21 (Figure 2C).

The ANOVA of SDS sedimentation volumes and protein content for gluten strength measured by the SDS sedimentation test considering allelic variation at Glu-A1, Glu-B1, and Glu-B3 loci as sources of variation are shown in Table 3. The allelic variation at the Glu-A1 locus showed no significant influence on gluten quality in the examined genotypes. The allelic variation at the Glu-B1 and Glu-B3 loci had a highly significant effect on gluten quality due to the highly significant impact of Glu-B1 and Glu-B3 alleles (Table 3).

**Figure 3:**
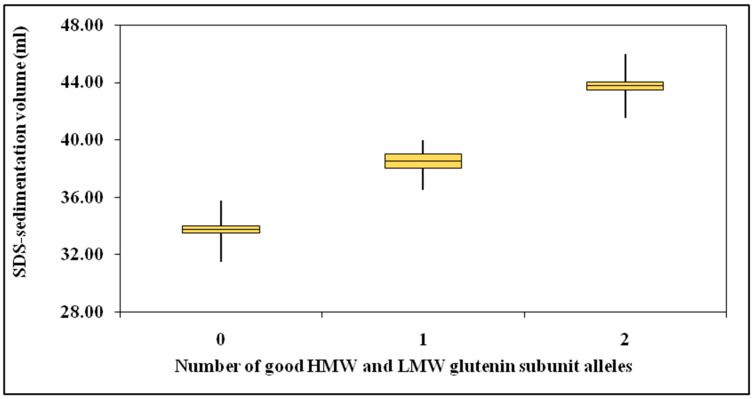
Cumulative effects of HMWGS and LMWGS alleles on gluten strength.

The combined better alleles atGlu-B1 andGlu-B3 loci showed linear cumulative effects for dough strength (Figure 3). This proved the positive effects ofHMWGS coded by Glu-B1 locus andLMWGS coded by theGlu-B3 locus were largely additive, and their physical interaction was not significant (Table 3). Additional evidence for the additive effects of Glu-A1, Glu-B1, and Glu-B3 alleles was provided by the observation that the mean regression response (averages over genotypes) of gluten strength to the varying number of “good” HMWGS and LMWGS alleles (alleles that are associated with good pasta quality) at Glu-B1and Glu-B3 loci was uniformly linear (Figure 3). Linear increases in gluten strength per “good” HMWGS or LMWGS allele were observed. Regression of gluten strength onto the number of “good” contributing HMWGS and LMWGS alleles produced a correlation value of r = 0.923 (*p* < 0.001). The average contribution of each “good” HMWGS and LMWGS allele to sedimentation volume was 5.5 mL. These results indicated the existence of considerable additive genetic variance in gluten strength. These data are essential, indicating that allelic variations at the Glu-B1 and Glu-B3 loci significantly affect the gluten strength additive variation.

No significant differences were observed for the protein content in the main effects and interactions of Glu-A1, Glu-B1, and Glu-B3 alleles. Moreover, including protein content as a covariate in the ANOVA analysis did not influence the results. This demonstrated that the protein content did not affect the associations between HMWGSs and LMWGSs and gluten quality. A discriminant functional analysis was conducted to summarize this information (Table 4). The first discriminant function was related to SDS sedimentation values, whereas the second was correlated with protein content. The squared canonical correlation of the first function indicated that 94% of the total variability might be due to allelic differences between HMWGS and LMWGS genotypes. The significant eigenvalue of the first function further supports this speculation. The considerable value of Wilks’ lambda for the second function indicated that HMWGs and LMWGSs appeared to be non-significant. After removing the first discriminant function (function derived 1), the significantly large level showed that the remaining function contributedless to the variation between the HMWGS and LMWGS genotypes. Only genotypes possessing the LMW-2 allele in durum genotypes with different Glu-B1 and Glu-B3 alleles revealed highly significant estimates in the regression analysis of SDSS volumes on protein content (Table 5).

Ultimately, the remarkably high proportion of gluten strength variability of the genotypescan be explained by allelic diversity in HMWGSs and LMWGSs. These findings proved the crucial functions of particular gluten proteins concerning functional properties and the existence of major genes that can simplify breeding for cooking quality in durum wheat by conventional methods.

### 2.2. Phylogeny and Amino Acid Sequence Analysis

**Figure 4:**
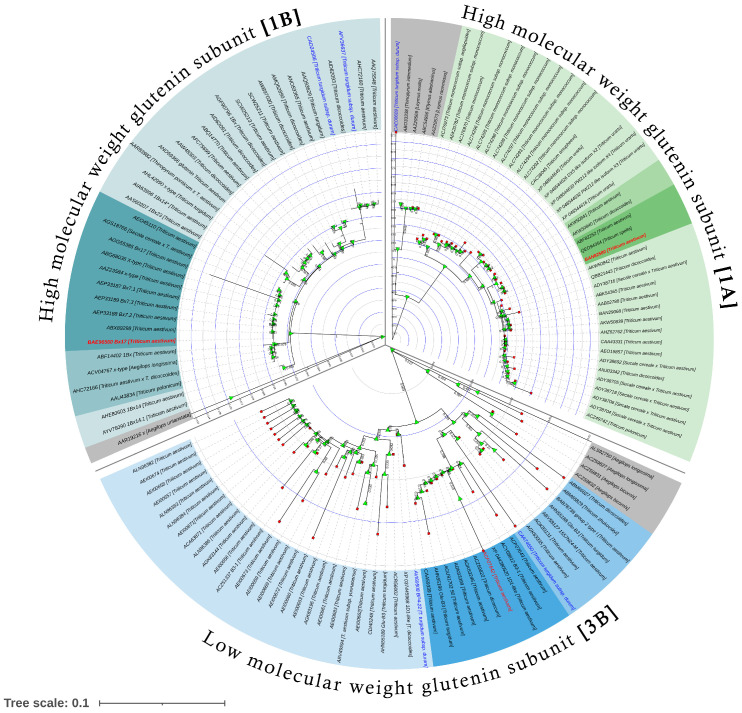
A phylogenetic tree showing the relationship between glutenin subunits from durum wheat, bread wheat, and their relative species. The tree was generated from the multiple sequence alignment of Glu-A1, Glu-B1, and Glu-B3 sequences in MEGA-X. The tree was rooted ontorye (*Secale cereale*) for the Glu-A1 subunit, and *Aegilops uniaristata* for the Glu-1B and Glu-B3 subunits. Bootstrap values were calculated from 1000 replications and only the values with 70% bootstrapping were considered significant, and are indicated on the branch nodes. The bread wheat sequences are indicated in red text, whereas the durum wheat sequences are indicated in blue text. The scale bar is shown at the bottom of the phylogenetic tree.

**Figure 5:**
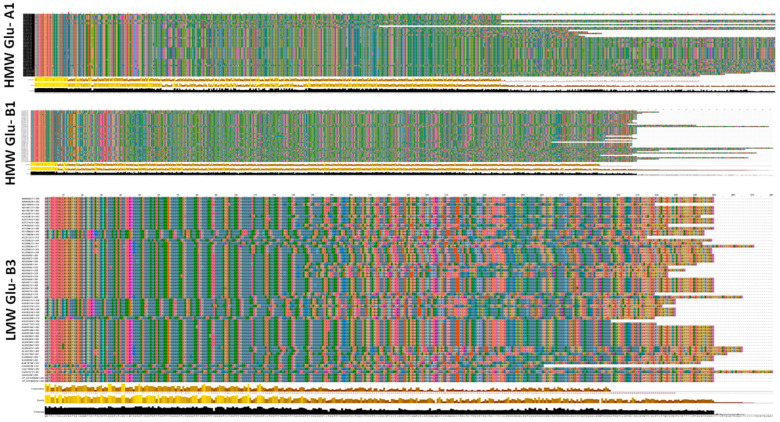
Multiple amino acid sequence alignments of HMWGS Glu-A1, Glu-B1, and Glu-B3 of durum wheat, bread wheat, and their relative species.

The amino acid sequences of Glu-A1, Glu-1 B, and Glu-B3 subunits of durum wheat and bread wheat genotypes were compared with their relative species (Figure 4, Figure 5 and Figures S1–S4). The Glu-A1 glutenin subunit of durum wheat showed the highest amino acid sequence similarity (79.0%) with the Glu-A1 glutenin subunit of *Leymusmollis* (AAZ29569) and the lowest similarity (71.95%) with *T. aestivum* (BAN2908) (Table S1). The Glu-B1glutenin subunit of durum wheat possessed low similarity with the Glu-B1 glutenin subunit of bread wheat and other relative species, ranging between 41.393% with *T. aestivum* [AAR19216] to 99.623% with *T. aestivum* [AHC72160]. The highest similarity (99.847%) (Table S2) appeared between *T. aestivum* [AEP33189] and *T. aestivum* [ADY38692]. The Glu-B3 glutenin subunit of durum wheat showed the highest amino acid sequence similarity (98.9%) with the Glu-B3 glutenin subunit of *T. aestivum* (CAA59339) and the lowest similarity (83%) with the Glu-B3 glutenin subunit of *T. turgidum* (CAD61021) (Table S3).

### 2.3. Determination of Repeat Motifs

The repeat domains of HMWGSs and LMWGSs were tri-, hexa-, and nona-peptide repeats (GQQ, PGQGQQ, and GYYPTSPQQ). The Glu-B1 subunit of *T. turgidum* contained31 tripeptide, 15 hexapeptide, and 8 nonapeptide domains, while these domains were absent in the amino acid sequence of Glu-A1 and Glu-B3 subunits (Table 6). On the other side, in *T. aestivum* the amino acid sequences of Glu-A1 and Glu-B1 subunits contained the three types of domains, while these domains were absent in the Glu-B3 subunit (Table 6).

### 2.4. Computational Chemical Analysis

#### 2.4.1. Glu-A1 Subunit

**Figure 6:**
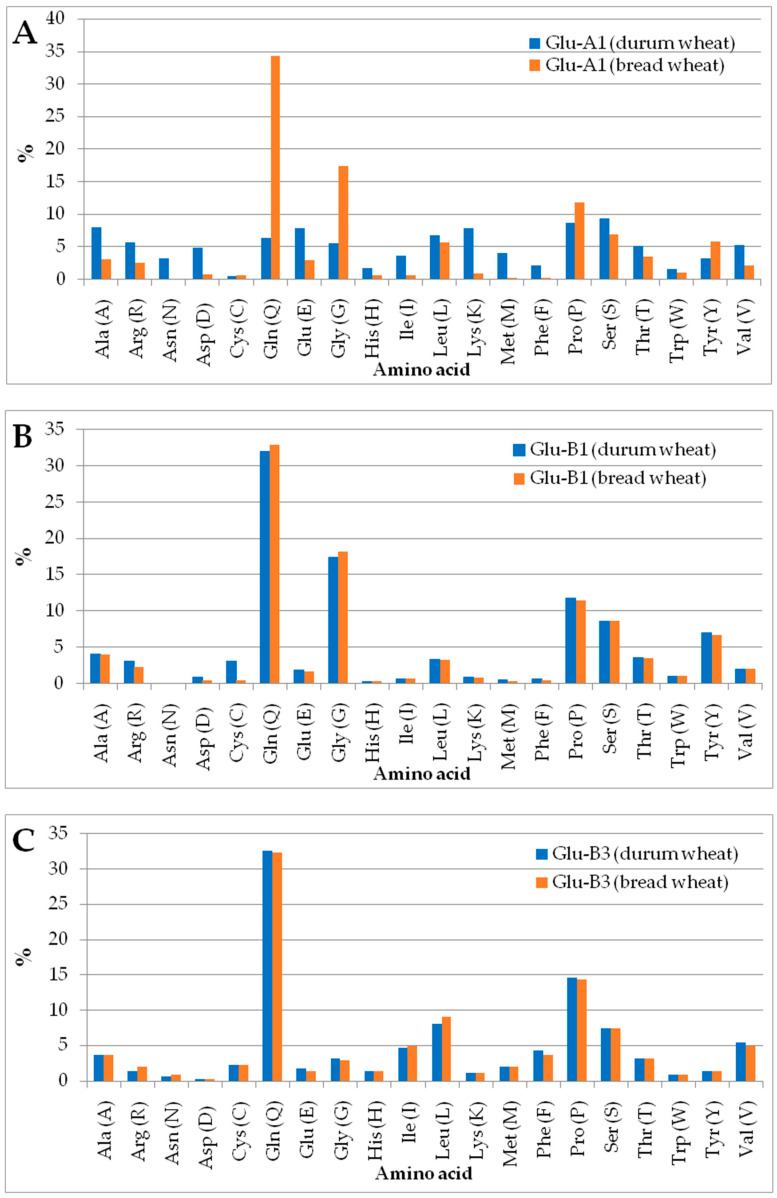
Amino acid composition of the Glu-A1 (**A**), Glu-B1 (**B**), and Glu-B3 (**C**) of durum and bread wheat.

The percentage of amino acid residues of the Glu-A1 glutenin subunit of durum wheat was compared with the Glu-A1 glutenin subunit of bread wheat (Figure 6A). The comparison showed that all amino acid content of the Glu-A1 glutenin subunit of durum wheat was higher than that of the Glu-A1 glutenin of bread wheat, except the glutamine, proline, glycine, and tyrosine amino acids. The computational analysis of protein parameters indicated that the negatively (Asp + Glu) and positively (Arg + Lys) charged residues in the Glu-A1 subunit of durum wheat were more significant (107 and 113) than those of bread wheat (29 and 28) (Table 7). The analysis also revealed that the Glu-A1subunit of durum wheatpossesses a higher aliphatic index (63.11), theoretical pI (8.66), and lower instability index (55.87) than the Glu-A1subunit of bread wheat (32.68), (6.54), and (94.49), respectively.

#### 2.4.2. Glu-B1 Subunit

The comparison between the amino acid residues of the Glu-B1 glutenin subunit of durum wheat and bread wheat showed that the former contained a higher content of all amino acids, except the glutamine, glycine, serine, and valine residues (Figure 6B). The analysis indicated that the Glu-B1 subunit of bread wheat contains a significant higher number of negatively and positively charged residues (20 and 31) than that of durum wheat (17 and 23) (Table 7). The analysis also revealed that the Glu-B1 subunit of durum wheat possesses higher a theoretical pI (9.21) andinstability index (91.64), and lower aliphatic index (24.70), than the Glu-B1 subunit of bread wheat ((8.81), (90.97), and (25.89), respectively).

#### 2.4.3. Glu-B3 Subunit

The comparison between the amino acid residues of the Glu-B3 glutenin subunit of durum wheat and bread wheat showed that the Glu-B3 of durum wheat includes a higher content of all amino acids, except the arginine, isoleucine, and leucine residues (Figure 6C). The analysis indicated that the Glu-B3 subunit of bread wheat contained seven negatively charged residues and nine positively charged residues, while durum wheat contained six negatively charged residues and eleven positively charged residues (Table 6). The Glu-B3 subunit of durum wheat possesses a higher aliphatic index (68.49), theoretical pI (8.14), and lower instability index (113.15) than the Glu-B3 subunit of bread wheat ((72.40), (8.87), and (111.91), respectively) (Table 7).

## 3. Discussion

Under similar growing conditions, durum wheat produces a lower yield and higher protein content than bread wheat [21]. The most investigated protein in wheat grain is glutenin because of its association with quality characteristics in wheat [9,22]. The SDS-sedimentation test has been widely utilized to determine the gluten strength in both durum and bread wheat [11]. A high sedimentation volume is directly linked to the quality of bread making [23]. Results indicated that the bread wheat had higher SDS sedimentation volumes than the durum wheat [23]. Moreover, previous studies showed a positive correlation between dough strength properties and the content of HMWGSs [24,25]. These results support the evidence that the main determinants of gluten elasticity are the HMWGSs. The current study proved that Glu-A1 and Glu-B1glutenin subunits were associated with a higher sedimentation volume. More specifically, the Glu-A1 glutenin subunit was associated with a higher sedimentation volume than the null subunit. Moreover, the study of the HMWGSs showed that genotypes possessing the subunits 7+8, 7+9, 17+18, or 13+16 had higher SDS-sedimentation volumes than those containing the subunits 7, 6+8, 20, 14+15, 13+19, or 21. The current result corroborates several studies that discussed the association between dough quality and the presence of HMWGSs and/or LMWGSs in durum and bread wheat [26,27,28,29].

In the tested set of durum wheat, allelic variation at the Glu-A1 locus had no detectable effect on the gluten quality. This lack of significance may be attributable to the higher proportion of null alleles (96%) and correspondingly less allelic variability at this locus. Only two genotypes within the HMWGS group displayed a null allele at the Glu-A1 locus, which is generally normal because most genotypes of modern durum wheat often show the null allele at the Glu-A1 locus [30,31,32]. The main effect of Glu-B1 and Glu-B3 alleles was highly significant, indicating that the properties of gluten were strongly associated with the variation at the Glu-B3 locus, and to a smaller extent, by allelic variation at Glu-B1. The obtained results agree with the previous studies of the effect of Glu-B1and Glu-B3 on gluten quality in wheat [15,33,34]. Nevertheless, Du Cros [18] found no consistent relationships between the gluten quality and the existence of specific HMWGSs in durum wheat. This may be due to the differential effects of HMWGS on gluten quality depending on their genetic background.

The linear cumulative effects for dough strength demonstrated by combining better alleles at the Glu-B1and Glu-B3 loci indicated the positive additive effects of HMWGSs. Consequently, genotypes containing HMWGS 7+8, 7+9, 13+16, 17+18, and LMWG-2, exhibited the best gluten properties. Such additivity between LMWGS and HMWGS alleles was reported by Gupta et al. [35] and Khoshro et al. [36] in bread wheat, and recently by Roncallo et al. [37] in durum wheat. The non-significant differences in the main effects and interactions of Glu-A1, Glu-B1, and Glu-B3 alleles for protein content indicated that the protein content didnot affect the associations observed between the gluten quality and the LMWGSs and HMWGSs. The weak correlation between the protein content and sedimentation volume in the experimental material strengthened the view that the sedimentation test is suitable to measure protein quality (i.e., protein type) rather than differences in protein content. Pena et al. [12] found that the protein content was not associated with the SDS sedimentation test.

The regression analysis of SDS sedimentation volumes on the protein content, evaluated in durum wheat genotypes possessing different alleles at Glu-B1 and Glu-B3 loci, indicated highly significant estimates only in genotypes including the LMW-2 allele. Ciaffi et al. [16] also reported a high correlation between the protein content and SDS sedimentation volume in durum wheat genotypes containing LMW-2 and γ-gliadin 45. In contrast, no association was found between the protein content and the SDS-sedimentation volumes in cultivars containing γ-gliadin 42 and LMW-1. This particular association could be due to the higher expression of the LMW-2 allele than the LMW-1 allele [14]. These findings showed that the increase in the protein content does not necessarily translate into good gluten strength unless specific LMWGS are present.

In silicoanalysis of HMWGSs and LMWGSsis of great importance for a better understanding of their relationship and similarity with glutenin subunits in the related species. The HMWGSs in many wheat-related species have been characterized and indexed in biological databases [38,39]. The use of bioinformatics tools to align and analyze multiple glutenin subunit sequences advances our knowledge of these proteins’ basic parameters, features, and aspects. The repeated motifs in HMWGSs may explain the molecular basis of their role in gluten functionality [40]. Shewry et al. [41] found that the central repeated motifs have hydrophilic characteristics, while the N- and C-terminal domains have a hydrophobic nature. Moreover, these motifs are expected to adopt a β-turn conformation [42].

Computational analysis revealed that the HMWGSs and LMWGSs contained higher proportions of glutamine, glycine, proline, serine, and tyrosine residues than the other residues. Interestingly, the Glu-A1 glutenin of durum wheat possessed a higher content of all amino acids than bread wheat, except glutamine, proline, glycine, and tyrosine. This result may explain the preference for durum wheat in pasta production due to its high elasticity-related content. In the same context, the Glu-B1 glutenin of durum wheat possessed a higher content of all amino acids than bread wheat, except glutamine, glycine, serine, and valine. These results revealed the importance of glutamine, proline, glycine, tyrosine, serine, and valine in the HMWGSs (Glu-A1 and Glu-B1) and arginine, isoleucine, and leucine in the LMWGSs (Glu-B3) in bread-making properties in bread wheat, and explain the suitability of durum wheat for pasta production. Ram et al. [43] reported that LMWGS glutenin showed high proline and glutamine residues. A particular feature of glutenin subunits is cysteine, which is responsible for dough elasticity. This residue aids in creating disulfide bonds between various glutenin and gliadin subunits, producing a good dough elasticity and supporting the suitability of durum wheat for pasta making. Bhatnagar et al. [44] reported that disulfide bonding involves the three conserved cysteine residues in the N-terminal of glutenin subunits. Interestingly, more cysteine residues were observed in Glu-B1 of durum wheat than in Glu-B1 of bread wheat.

In addition to the importance of disulfide bonds for stabilizing the HMWGSs, another important factor in maintaining the stability of the glutenin structure may be the hydrogen bonding mediated by glutamine side chains [45]. The average of other computed protein parameters, negatively and positively charged residues, aliphatic index, and theoretical pI of the glutenin of durum wheat were higher than those in breadwheat. The high aliphatic index indicates that the glutenins of durum wheat are more thermo-stable over a wide temperature range than the glutenins of bread wheat [46]. Although the instability index of the Glu-B3 subunit in durum wheat showed the highest value, it is predicted that it is more stable in durum wheat than in bread wheat. All parameters of the Glu-B1 subunit were higher in durum wheat than in bread wheat except the aliphatic index. All parameters of the Glu-B3 subunit were lower in durum wheat than in bread wheat except the negatively charged (Asp + Glu) residues and the instability index. The presence of negatively and positively charged residues promotes interactions and folding of the glutenins [47]. This comparison of parameters could be helpful in distinguishing the dough quality and elasticity. Plant breeders should take HMWGS composition into account when choosing parents for crosses intended to produce lines of good baking quality. The parental varieties should have complementary good-quality glutenin subunits, so that they can be combined in a few progeny that will have better quality than that of their parents. The detection of such associations, as reported here, is also of significance to the development of early generation selection strategies. SDS-gel electrophoresis can be used in the breeding program of fairly early generations as a secondary screen to select the few progenythat have the desired protein composition for a particular cross.

This knowledge will also be exploited in screening for good-quality glutenin subunits from diverse sources, including landraces of ancient agriculture and wild diploid species related to wheat. The genes coding for them would then be transferred to commercial varieties by recurrent backcrossing procedures.

## 4. Materials and Methods

### 4.1. Plant Materials and Experiment Design

The current study included 51 tetraploid durum wheat genotypes. The evaluated genotypes were obtained from CIMMYT and ICARDA to evaluate the association between HMWGSs and LMWGSs for gluten strength.

The seeds were planted in three replicates ina randomized complete block design (RCBD) at the experimental farm of the Faculty of Agriculture, Zagazig University (Latitude: 30°35′15″ N, Longitude: 31°30′07″ E, Elevation above sea level: 16 m = 52 ft), in the growing season 2020/2021. According to the recommended period of wheat cultivation in the study region, sowing took place during the third week of November. The standard agronomic practices in the study region, including irrigation, fertilization, weed control, and pest control, were applied.

### 4.2. Protein Extraction and Gluten Strength Evaluation

Wheat grains from each genotype were milled using a Micro-Mill. Whole meal flour samples were then used to evaluate the strength of wheat gluten by the SDS sedimentation test according to Axford et al. [11] and as described by Lorenzo and Kronstad [48]. In this method, 5 g of flour samples was suspended in 100 mL of 2% SDS (*w*/*v*) and lactic acid (0.96 g/L), and the precipitate volume was measured. The flour samples with high SDS sedimentation volumes were considered for the presence of strong gluten. The protein content inthewholemeal was determined according to the Kjeldahl method [49].

### 4.3. SDS-PAGE

To investigate the association of the high and low molecular weight glutenin subunits with the gluten strength in durum wheat, the total endosperm storage proteins were extracted from wheat grains using 2% (*w*/*v*) sodium dodecyl sulfate (SDS), 6 M urea, and 1.5% (*v*/*v*) 2-mercaptoethanol (2-ME). The extraction solution contained 0.002% (*w*/*v*) of bromophenol blue (tracking dye). Wheat grains were crushed and transferred to a 1.5 mL tube with 0.4 mL of the extracting solution and incubated overnight at room temperature. Extracts were used directly for electrophoresis.

The SDS-PAGE method used to separate the wheat storage proteins was adapted from that reported by Laemmli [50]. The separating gel (0.36 M Tris-HCL, pH 9.1 and 0.1% (*w*/*v*) SDS) consisted of 10% (*w*/*v*) acrylamide and 0.125% (*w*/*v*) Bis (N, N′-Methylenebisacrylamide). The stacking gel was made from 3% (*w*/*v*) acrylamide, 0.25% (*w*/*v*) Bis, 0.1% (*w*/*v*) SDS, and 0.006 M Tris-phosphate (pH 6.7). Ammonium persulfate and TEMED (N, N, N′, N′-tetramethylethylenediamine) was used to polymerize the gels. The gels were photographed using the Bio-Rad Gel Doc EZ Gel documentation system. The scoring system used for the HMWGS and allele classification at the Glu-1 loci was described by Payne and Lawrence [26]. LMWGS were designated according to Payne et al. [14]. Subunit designations such as 7+8 and LMW-1 refer to their coding alleles and subunit combinations.

### 4.4. Bioinformatics Analysis

The Glu-A1, Glu-B1, and Glu-B3 amino acid sequences of durum wheat, bread wheat, and their closely related genotypes were retrieved from the NCBI GenBank database using the NCBI BLAST service using a similarity search. After manual organization, the sequences were subjected to multiple amino acid sequence alignment using the CLUSTAL-W tool in MEGA-X software [51]. The phylogenetic trees were constructed in the Clustal-X software Neighbor-Joining (NJ) algorithm using the multiple sequence alignment file of the amino acid sequences [52].

### 4.5. Computational Chemical Analysis

To understand the association of the content of glutamine, proline, glycine, tyrosine, serine, and valine in the Glu-A1 and Glu-B1 glutenin subunits, the content of cysteine residues in Glu-B1, and the content of arginine, isoleucine, and leucine in the Glu-B3 glutenin, with the suitability of durum wheat for pasta making and the suitability of bread wheat with good bread-making quality, the ProtParam tool (https://web.expasy.org/protparam/ (accessed on 5 April 2007)) was used for in silico computing of the chemical parameters of Glu-A1, Glu-B1, and Glu-B3 glutenins. The computed properties included the amino acid composition, protein molecular weight, aliphatic index (alanine, valine, isoleucine, and leucine), theoretical isoelectric point (pI), negatively and positively charged residues, hydrophobicity index, and instability index [53].

### 4.6. Statistical Analysis

Analysis of variance and discriminant function analysis were employed for SDS-sedimentation and protein content using R software version 4.1.1. The box and whisker plots for allelic variation and gluten strength were constructed using Excel (Microsoft Office 365) version 1903 (Build 11425.20202)

## 5. Conclusions

This study indicated that SDS-PAGE was proven to be a successful method for determining the HMWGS and LMWGS alleles and their association with the dough quality. The genotypes containing the HMWGS alleles 7+8, 7+9, 13+16, and 17+18 were highly associated with improved dough strength. The LMW-2 allele was associated with stronger gluten than the LMW-1 allele. The comparative in silico analysis indicated that the Glu-A1 and Glu-B1 glutenin subunits possessed high contents of serine and valine, and low contents of glutamine, proline, glycine, and tyrosine, while the Glu-B1 glutenin subunit possessed high contents of cysteine residues. The Glu-B3 glutenin subunit possessed low contents of arginine, isoleucine, and leucine residues, which are associated with the suitability of bread wheat for good bread-making quality and durum wheat for pasta making. The phylogeny analysis reported that both Glu-B1 and Glu-B3 had a closer evolutionary relationship in bread and durum wheat, while Glu-A1 was highly distinct. The results of the current research may help breeders to manage the quality of durum wheat genotypes by exploiting the allelic variation in glutenin, and to consider the association of HMWGS and LMWGS glutenin subunits with dough quality properties.

## Figures and Tables

**Table 1:** Genotypic classes of different HMWGSs and LMWGSs as revealed by SDS-PAGE, and their mean values of protein content and SDSS testing.

Glutenin Subunit	HMWGS and LMWGS			N^a^	Frequency (%)	Mean (±SE) Value for	
	Glu-A1	Glu-B1	Glu-B3			Protein Content (%)	SDSS Volume (mL)
Genotypic Class							
1	1	7+9	LMW-2	1	1.96	14.32	45.25
2	1	13+16	LMW-2	1	1.96	12.98	41.25
3	Null	7	LMW-1	7	13.73	13.22 ± 0.26	33.32 ± 0.79
4	Null	7	LMW-2	3	5.88	13.26 ± 0.25	38.92 ± 1.45
5	Null	7+8	LMW-1	1	1.96	14.23	36.5
6	Null	7+8	LMW-2	3	5.88	14.32 ± 0.42	46.25 ± 0.29
7	Null	7+9	LMW-2	3	5.88	14.11 ± 0.28	45.08 ± 1.09
8	Null	6+8	LMW-1	5	9.80	14.18 ± 0.18	33.30 ± 0.43
9	Null	6+8	LMW-2	3	5.88	13.74 ± 0.50	37.75 ± 0.95
10	Null	20	LMW-1	3	5.88	14.03 ± 0.33	35.42 ± 0.51
11	Null	20	LMW-2	3	5.88	13.88 ± 0.26	39.42 ± 1.17
12	Null	13+16	LMW-1	5	9.80	13.58 ± 0.34	39.80 ± 0.18
13	Null	13+16	LMW-2	6	11.76	14.23 ± 0.24	45.08 ± 0.95
14	Null	13+19	LMW-2	3	5.88	13.57 ± 0.38	36.50 ± 1.23
15	Null	14+15	LMW-1	1	1.96	13.75	31.25
16	Null	17+18	LMW-2	2	3.92	13.26 ± 0.31	40.75 ± 0.50
17	Null	21	LMW-1	1	1.96	14.32	32.25

**N^a^** Number of durum wheat in each glutenin subunit, the full genotype list was in Table S1.

**Table 2:** Mean values for protein content (%) and SDS sedimentation volumes for HMWGS and LMWGS alleles at Glu-A1, Glu-B1, and Glu-B3 loci.

Allelic Group	N^a^	Mean (±SE) Value	
		Protein Content (%)	SDSS Volume (mL)
*Glu-A1alleles*			
1	2	13.65 ± 0.67	43.25 ± 2.00
null	49	13.80 ± 0.09	38.55 ± 0.70
*Glu-B1alleles*			
7	10	13.23 ± 0.18	35.00 ± 1.08
7+8	4	14.30 ± 0.30	43.81 ± 2.44
7+9	4	14.16 ± 0.20	45.13 ± 0.77
6+8	8	14.01 ± 0.21	34.97 ± 0.90
20	6	13.96 ± 0.19	37.42 ± 1.06
13+16	12	13.85 ± 0.21	42.56 ± 0.89
13+19	3	13.57 ± 0.38	36.50 ± 1.28
14+15	1	13.75	31.25
17+18	2	13.26 ± 0.31	40.75 ± 0.50
21	1	14.32	32.25
*Glu-B3 alleles*			
LMW-1	24	13.69 ± 0.14	35.26 ± 0.66
LMW-2	27	13.88 ± 0.12	41.82 ± 0.77

**N^a^** Number of durum wheat in each allelic group.

**Table 3:** Analysis of variance (ANOVA) for the protein content and SDS volumes with main effects of HMWGS and LMWGS loci and their two-way interactions.

Source of Variation	DF	Mean Square	
		SDS-Sedimentation	Protein Content
Glu-A1.(A)	1	1.75	0.30
Glu-B1.(B)	9	45.06 ***	0.74
Glu-B3.(C)	1	76.13 ***	1.15
A × B	1 ^a^	0.65	0.26
B × C	4 ^b^	4.37	0.37
Error	34	3.03	0.39

*** indicates *p*-value < 0.001, ^a^ Eight2-locus combinations (1 at Glu-A1with each of 7, 7+8, 6+8, 20, 13+19, 14+15, 17+18 and 21 at Glu-B1 locus) were not present in this set of genotypes; therefore, this source has 1 instead of 9 degrees of freedom. ^b^ Five 2-locus combinations (LMW-1 with 7+9, 13+19 and 17+18, and LMW-2 with 14+15 and 21) were absent; therefore, this source has 4 instead of 9 degrees of freedom.

**Table 4:** Discriminant function analysis of durum wheat genotypesconsidering different HMWGS and LMWGS genotypes as groups.

**Standardized Canonical Discriminant Function Coefficients**							
	**1**			**2**			
Protein content	−0.03602			1.00824			
SDSS test	1.00413			−0.09779			

**Discriminant Function**	**Eigenvalue Percentage**		**Relative Correlation**				**Canonical**
1	10.7024		94.98				0.9563
2	0.5651		5.02				0.6009

**FunctionDerived**	**Wilks’Lambda**	**Chi-Square**			**DF**	***p*-Value**	
0	0.054598	117.764			32	0.0000	
1	0.638926	18.143			15	0.2552	

**Table 5:** Analysis of variance for regression of SDSS volumes on protein content in genotypes grouped based on the Glu-B3 alleles.

SOV	DF	Mean Square
***LMW-1 allele***		
Regression.	1	8.644
Residual.	22	10.683
***LMW-2 allele***		
Regression.	1	135.606 **
Residual.	25	11.244

** indicates *p*-value > 0.001.

**Table 6:** Repeat motifs of Glu-A1, Glu-B1, and Glu-B3 of *T. turgidum* and *T. aestivum*.

Motif	*T. turgidum*			*T. aestivum*		
	Glu-A1	Glu-B1	Glu-B3	Glu-A1	Glu-B1	Glu-B3
Tripeptide (GQQ)	0	31	0	41	40	0
Hexapeptide (PGQGQQ)	0	15	0	7	14	0
Nona peptide (GYYPTSPQQ)	0	8	0	6	9	0

**Table 7:** The computed protein parameters: protein size, molecular weight, extinction coefficient, and atomic composition of Glu-A1, Glu-B1, and Glu-B3 of *Triticum turgidum* and *Triticum aestivum*.

	*Triticum turgidum*			*Triticum aestivum*		
	Glu-A1	Glu-B1	Glu-B3	Glu-A1	Glu-B1	Glu-B3
Protein size (aa)	846	795	350	824	747	350
Protein MW (KD)	87.29	86.07	39.82	89.45	80.14	39.91
(Asp + Glu)	107	20	7	29	17	6
(Arg + Lys)	113	31	9	28	23	11
Aliphatic index	63.11	24.70	68.49	32.68	25.89	72.40
Instability index	55.87	91.64	113.15	94.49	90.97	111.91
Theoretical pI	8.66	9.21	8.14	6.54	8.81	8.87

## Data Availability

The data presented in this study are available upon request from the corresponding authors.

## Supplementary Materials

The following supporting information can be downloaded at: https://www.mdpi.com/article/10.3390/plants12061416/s1, Figure S1. phylogenetic tree showing the relationship between glutenin subunits from durum wheat, bread wheat, and their relative species; Figure S2. Multiple amino acid sequence alignments of HMWGS Glu-A1 of durum wheat, bread wheat, and their relative species; Figure S3. Multiple amino acid sequence alignments of HMWGS Glu-B1 of durum wheat, bread wheat, and their relative species; Figure S4. Multiple amino acid sequence alignments of HMWGS Glu-B3 of durum wheat, bread wheat, and their relative species; Table S1. Different HMWGS and LMWGS as revealed by SDS-PAGE; protein content (%) and SDSS volume (mL).

## References

[1] Saini P., Kaur H., Tyagi V., Saini P., Ahmed N., Dhaliwal H., Sheikh I. (2022). Nutritional value and end-use quality of durum wheat. Cereal Res. Commun..

[2] Weegels P., Van de Pijpekamp A., Graveland A., Hamer R., Schofield J. (1996). Depolymerisation and re-polymerisation of wheat glutenin during dough processing. I. Relationships between glutenin macropolymer content and quality parameters. J. Cereal Sci..

[3] Shewry P.R., Tatham A.S., Lazzeri P. (1997). Biotechnology of wheat quality. J. Sci. Food Agric..

[4] Yu Z., Peng Y., Islam M.S., She M., Lu M., Lafiandra D., Roy N., Juhasz A., Yan G., Ma W. (2019). Molecular characterization and phylogenetic analysis of active y-type high molecular weight glutenin subunit genes at Glu-A1 locus in wheat. J. Cereal Sci..

[5] Guzmán C., Crossa J., Mondal S., Govindan V., Huerta J., Crespo-Herrera L., Vargas M., Singh R.P., Ibba M.I. (2022). Effects of glutenins (Glu-1 and Glu-3) allelic variation on dough properties and bread-making quality of CIMMYT bread wheat breeding lines. Field Crops Res..

[6] Singh N., Shepherd K., Cornish G. (1991). A simplified SDS-PAGE procedure for separating. J. Cereal Sci..

[7] Carrillo J., Martinez M., Brites C., Nieto-Taladriz M., Vázquez J. (2000). Relationship between endosperm proteins and quality in durum wheat (*Triticum turgidum* L. var. durum). Options Mediterr..

[8] Shewry P.R., Halford N.G., Tatham A.S. (1992). High molecular weight subunits of wheat glutenin. J. Cereal Sci..

[9] Nazco R., PeÑA R.J., Ammar K., Villegas D., Crossa J., Moragues M., Royo C. (2013). Variability in glutenin subunit composition of Mediterranean durum wheat germplasm and its relationship with gluten strength. J. Agric. Sci..

[10] Singh N., Shepherd K. (1985). The structure and genetic control of a new class of disulphide-linked proteins in wheat endosperm. Theor. Appl. Genet..

[11] Axford D.W.E., McDermott E.E., Redman D.G. (1979). Note on the sodium dodecyl sulphate sedimentation test and bread-making quality: Comparison with Pelshenke and Zeleny tests. Cereal Chem..

[12] Pena R., Zarco-Hernandez J., Mujeeb-Kazi A. (1995). Glutenin subunit compositions and breadmaking quality characteristics of synthetic hexaploid wheats derived from *Triticum turgidum* × *Triticum tauschii* (coss.) Schmal Crosses. J. Cereal Sci..

[13] Damidaux R., Autran J.C., Grignac P., Feillet P. (1978). Mise enévidence de relations applicables en sélection entre l′électrophorégramme des gliadines et les proprieties viscoé-lastiques du gluten de Triticum durum Desf. associées a la qualitéculinarieintrinséque des variétés. CR Acad. Sci. Paris Ser. D..

[14] Payne P.I., Jackson E.A., Holt L.M. (1984). The association between γ-gliadin 45 and gluten strength in durum wheat varieties: A direct causal effect or the result of genetic linkage?. J. Cereal Sci..

[15] Pogna N., Autran J.-C., Mellini F., Lafiandra D., Feillet P. (1990). Chromosome 1B-encoded gliadins and glutenin subunits in durum wheat: Genetics and relationship to gluten strength. J. Cereal Sci..

[16] Ciaffi M., Benedettelli S., Giorgi B., Porceddu E., Lafiandra D. (1991). Seed storage proteins of Triticum turgidum ssp. dicoccoides and their effect on the technological quality in durum wheat. Plant Breed..

[17] Sharma A., Garg S., Sheikh I., Vyas P., Dhaliwal H. (2020). Effect of wheat grain protein composition on end-use quality. J. Food Sci. Technol..

[18] Du Cros D. (1987). Glutenin proteins and gluten strength in durum wheat. J. Cereal Sci..

[19] Dreisigacker S., Xiao Y., Sehgal D., Guzman C., He Z., Xia X., Pena R.J. (2020). SNP markers for low molecular glutenin subunits (LMW-GSs) at the Glu-A3 and Glu-B3 loci in bread wheat. PLoS ONE.

[20] Hernández-Espinosa N., Payne T., Huerta-Espino J., Cervantes F., Gonzalez-Santoyo H., Ammar K., Guzmán C. (2019). Preliminary characterization for grain quality traits and high and low molecular weight glutenins subunits composition of durum wheat landraces from Iran and Mexico. J. Cereal Sci..

[21] Branković G., Dodig D., Pajić V., Kandić V., Knežević D., Djurić N., Živanović T. (2018). Genetic parameters of Triticum aestivum and Triticum durum for technological quality properties in Serbia. Zemdirb.-Agric..

[22] Sissons M.J., Ames N.P., Hare R.A., Clarke J.M. (2005). Relationship between glutenin subunit composition and gluten strength measurements in durum wheat. J. Sci. Food Agric..

[23] Dexter J., Preston K., Martin D., Gander E. (1994). The effects of protein content and starch damage on the physical dough properties and bread-making quality of Canadian durum wheat. J. Cereal Sci..

[24] Field J., Tatham A., Shewry P. (1987). The structure of a high-M r subunit of durum-wheat (*Triticum durum*) gluten. Biochem. J..

[25] Anjum F.M., Lookhart G.L., Walker C.E. (2000). High-molecular-weight glutenin subunit composition of Pakistani hard white spring wheats grown at three locations for 2 years and its relationship with end-use quality characteristics. J. Sci. Food Agric..

[26] Payne P.I., Lawrence G.J. (1983). Catalogue of alleles for the complex gene loci, Glu-A1, Glu-B1, and Glu-D1 which code for high-molecular-weight subunits of glutenin in hexaploid wheat. Cereal Res. Commun..

[27] Dong H., Cox T., Sears R., Lookhart G. (1991). High molecular weight glutenin genes: Effects on quality in wheat. Crop Sci..

[28] Czuchajowska Z., Lin P., Smolinski S. (1996). Role in dough rheology of high molecular weight glutenin subunits of soft white winter and club wheats. Cereal Chem..

[29] Peña R., Zarco-Hernandez J., Amaya-Celis A., Mujeeb-Kazi A. (1994). Relationships between chromosome 1B-encoded glutenin subunit compositions and bread-making quality characteristics of some durum wheat (*Triticum turgidum*) cultivars. J. Cereal Sci..

[30] Ammar K., Kronstad W.E., Morris C.F. (2000). Breadmaking Quality of Selected Durum Wheat Genotypes and Its Relationship with High Molecular Weight Glutenin Subunits Allelic Variation and Gluten Protein Polymeric Composition. Cereal Chem..

[31] Henkrar F., El-Haddoury J., Iraqi D., Bendaou N., Udupa S.M. (2017). Allelic variation at high-molecular weight and low-molecular weight glutenin subunit genes in Moroccan bread wheat and durum wheat cultivars. 3 Biotech.

[32] Magallanes-López A.M., Ammar K., Morales-Dorantes A., González-Santoyo H., Crossa J., Guzmán C. (2017). Grain quality traits of commercial durum wheat varieties and their relationships with drought stress and glutenins composition. J. Cereal Sci..

[33] Izadi-Darbandi A., Yazdi-Samadi B., Shanejat-Boushehri A.-A., Mohammadi M. (2010). Allelic variations in Glu-1 and Glu-3 loci of historical and modern Iranian bread wheat (*Triticum aestivum* L.) cultivars. J. Genet..

[34] Pandey V., Kapoor S., Patwa N., Gupta O.P., Gopalareddy K., Ram S., Singh G.P. (2022). Molecular, Biotechnological and Omics-Based Interventions for Improving Wheat Grain Quality: Advances and Way Forward. New Horizons in Wheat and Barley Research.

[35] Gupta R., Singh N., Shepherd K. (1989). The cumulative effect of allelic variation in LMW and HMW glutenin subunits on dough properties in the progeny of two bread wheats. Theor. Appl. Genet..

[36] Khoshro H.H., Bihamta M.R., Hassani M.E. (2022). Relationship between allelic variation at the Glu-3 loci and qualitative traits in bread wheat. Cereal Res. Commun..

[37] Roncallo P.F., Guzmán C., Larsen A.O., Achilli A.L., Dreisigacker S., Molfese E., Astiz V., Echenique V. (2021). Allelic variation at glutenin loci (Glu-1, Glu-2 and Glu-3) in a worldwide durum wheat collection and its effect on quality attributes. Foods.

[38] Liu Z., Yan Z., Wan Y., Liu K., Zheng Y., Wang D. (2003). Analysis of HMW glutenin subunits and their coding sequences in two diploid Aegilops species. Theor. Appl. Genet..

[39] Wang J.-R., Yan Z.-H., Wei Y.-M., Zheng Y.-L. (2004). A novel high-molecular-weight glutenin subunit gene Ee1. 5 from Elytrigiaelongata (Host) Nevski. J. Cereal Sci..

[40] Shewry P.R., Field J.M., Faulks A.J., Parmar S., Miflin B.J., Dietler M.D., Lew E.J., Kasarda D.D. (1984). The purification and N-terminal amino acid sequence analysis of the high molecular weight gluten polypeptides of wheat. Biochim. Biophys. Acta (BBA)-Protein Struct. Mol. Enzymol..

[41] Shewry P., Halford N., Tatham A. (1989). The high molecular weight subunits of wheat, barley and rye: Genetics, molecular biology, chemistry and role in wheat gluten structure and fuctionality. Oxford Surveys of Plant Molecular and Cell Biology.

[42] Tatham A.S., Drake A.F., Shewry P.R. (1990). Conformational studies of synthetic peptides corresponding to the repetitive regions of the high molecular weight (HMW) glutenin subunits of wheat. J. Cereal Sci..

[43] Ram S., Bhatia V., Jain V., Mishra B. (2006). Characterization of Low Molecular Weight Glutenin Subunit Gene Representing Glu-B3 Locus of Indian Wheat Variety NP4. J. Plant Biochem. Biotechnol..

[44] Bhatnagar T., Sachdev A., Johari R. (2002). Molecular characterization of glutenins in wheat varieties differing in chapati quality characteristics. J. Plant Biochem. Biotechnol..

[45] Belton P.S., Colquhoun I.J., Grant A., Wellner N., Field J.M., Shewry P.R., Tatham A.S. (1995). FTIR and NMR studies on the hydration of a high-Mr subunit of glutenin. Int. J. Biol. Macromol..

[46] Ikai A. (1980). Thermostability and aliphatic index of globular proteins. J. Biochem..

[47] Indrani D., Rao G.V. (2003). Influence of surfactants on rheological characteristics of dough and quality of parotta. Int. J. Food Sci. Technol..

[48] Lorenzo A., Kronstad W. (1987). Reliability of Two Laboratory Techniques to Predict Bread Wheat Protein Quality in Nontraditional Growing Areas 1. Crop Sci..

[49] Kjeldahl J. (1883). Neue methodezurbestimmung des stickstoffs in organischenkörpern. Z. Anal. Chem..

[50] Laemmli U.K. (1970). Cleavage of structural proteins during the assembly of the head of bacteriophage T4. Nature.

[51] Kumar S., Stecher G., Li M., Knyaz C., Tamura K. (2018). MEGA X: Molecular Evolutionary Genetics Analysis across Computing Platforms. Mol. Biol. Evol..

[52] Zhang W., Sun Z. (2008). Random local neighbor joining: A new method for reconstructing phylogenetic trees. Mol. Phylogenet. Evol..

[53] Walker J.M. (2005). The Proteomics Protocols Handbook.

