# Supplementary material for: Association of High and Low Molecular Weight Glutenin Subunits with Gluten Strength in Tetraploid Durum Wheat (Triticum turgidum spp. Durum L.)
Source: Plants (Basel). 2023 Mar 22;12(6):1416. doi: 10.3390/plants12061416 (PMC10051775; doi:10.3390/plants12061416)
Supplement: Supplementary file 1 [file plants-12-01416-s001.zip › Supplementary Table 1.docx]

**Table S1.** Different HMWGS and LMWGS as revealed by SDS-PAGE; protein content (%) and SDSS volume (ml).

| **Genotypes** | **Glutenin Subunit** | | |  | |
| --- | --- | --- | --- | --- | --- |
|  | **HMWGS and LMWGS** | | | **Protein content (%)** | **SDSS volume (ml)** |
|  | **.Glu-A1** | **.Glu-B1** | **.Glu-B3** |  |  |
|  |  |  |  |  |  |
| 1 | Null. | 20 | .LMW-1 | 28.50 | 14.85 |
| 2 | Null. | 6+8 | .LMW-1 | 30.75 | 13.95 |
| 3 | Null. | 20 | .LMW-2 | 32.75 | 14.35 |
| 4 | Null. | 6+8 | .LMW-1 | 33.25 | 15.15 |
| 5 | Null. | 13+16 | .LMW-2 | 43.50 | 13.95 |
| 6 | Null. | 13+16 | .LMW-2 | 45.75 | 13.65 |
| 7 | Null. | 17+18 | .LMW-2 | 41.25 | 12.60 |
| 8 | Null. | 13+16 | .LMW-1 | 39.75 | 13.65 |
| 9 | Null. | 20 | .LMW-1 | 32.25 | 14.15 |
| 10 | 1. | 13+16 | .LMW-2 | 45.25 | 12.85 |
| 11 | Null. | 13+16 | .LMW-1 | 39.75 | 13.75 |
| 12 | Null. | 13+16 | .LMW-1 | 40.50 | 11.95 |
| 13 | Null. | 7+9 | .LMW-2 | 44.75 | 14.20 |
| 14 | Null. | 13+19 | .LMW-1 | 39.50 | 13.25 |
| 15 | Null. | 13+19 | .LMW-2 | 38.50 | 14.35 |
| 16 | 1. | 7+9 | .LMW-2 | 46.25 | 12.65 |
| 17 | Null. | 20 | .LMW-2 | 37.25 | 13.45 |
| 18 | Null. | 13+16 | .LMW-2 | 48.75 | 13.85 |
| 19 | Null. | 20 | .LMW-2 | 38.75 | 14.35 |
| 20 | Null. | 14+15 | .LMW-1 | 31.25 | 13.75 |
| 21 | Null. | 13+16 | .LMW-2 | 43.25 | 13.15 |
| 22 | Null. | 7 | .LMW-1 | 35.25 | 12.75 |
| 23 | Null. | 13+16 | .LMW-2 | 43.75 | 14.35 |
| 24 | Null. | 7 | .LMW-1 | 34.25 | 13.60 |
| 25 | Null. | 7 | .LMW-1 | 37.75 | 14.65 |
| 26 | Null. | 7 | .LMW-1 | 39.50 | 12.90 |
| 27 | Null. | 7 | .LMW-2 | 35.75 | 13.45 |
| 28 | Null. | 6+8 | .LMW-1 | 34 | 14.10 |
| 29 | Null. | 13+19 | .LMW-2 | 38.50 | 12.65 |
| 30 | Null. | 7 | .LMW-2 | 39.25 | 11.95 |
| 31 | Null. | 7 | .LMW-2 | 41.25 | 13.25 |
| 32 | Null. | 7 | .LMW-1 | 37 | 14.05 |
| 33 | Null. | 7+9 | .LMW-2 | 44.25 | 14.35 |
| 34 | Null. | 20 | .LMW-1 | 36.25 | 13.95 |
| 35 | Null. | 6+8 | .LMW-1 | 35.50 | 15.10 |
| 36 | Null. | 6+8 | .LMW-1 | 37.25 | 13.85 |
| 37 | Null. | 6+8 | .LMW-1 | 33.25 | 14.15 |
| 38 | Null. | 6+8 | .LMW-2 | 38.50 | 13.65 |
| 39 | Null. | 17+18 | .LMW-2 | 40.25 | 12.95 |
| 40 | Null. | 7+8 | .LMW-2 | 45.25 | 13.85 |
| 41 | Null. | 13+16 | .LMW-1 | 41.25 | 14.25 |
| 42 | Null. | 13+16 | .LMW-2 | 47.50 | 13.90 |
| 43 | Null. | 6+8 | .LMW-1 | 35 | 12.75 |
| 44 | Null. | 7+9 | .LMW-1 | 31.50 | 14.35 |
| 45 | Null. | 7+8 | .LMW-2 | 45.75 | 15.05 |
| 46 | Null. | 20 | .LMW-1 | 34.25 | 14.30 |
| 47 | Null. | 7+8 | .LMW-1 | 36.50 | 14.25 |
| 48 | Null. | 7+8 | .LMW-2 | 41.50 | 13.65 |
| 49 | Null. | 13+19 | .LMW-2 | 38.50 | 12.85 |
| 50 | Null. | 7 | .LMW-1 | 35.75 | 11.95 |
| 51 | Null. | 7 | .LMW-1 | 33.25 | 13.55 |
