# Supplementary figures and images for: Association of High and Low Molecular Weight Glutenin Subunits with Gluten Strength in Tetraploid Durum Wheat (Triticum turgidum spp. Durum L.)
Source: Plants (Basel). 2023 Mar 22;12(6):1416. doi: 10.3390/plants12061416 (PMC10051775; doi:10.3390/plants12061416)

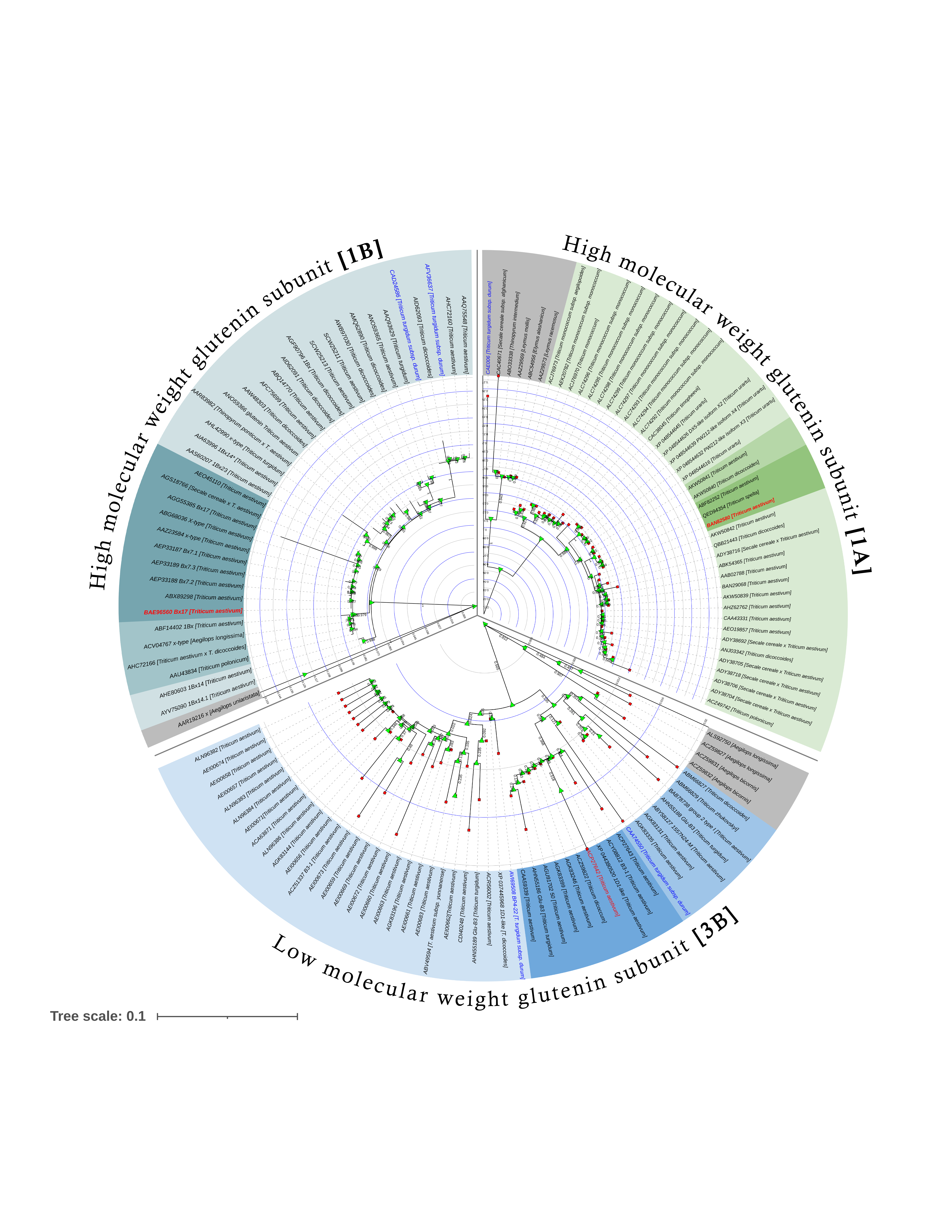

Supplement: Supplementary file 1 [file plants-12-01416-s001.zip › Supplementary Figure 1.tif]

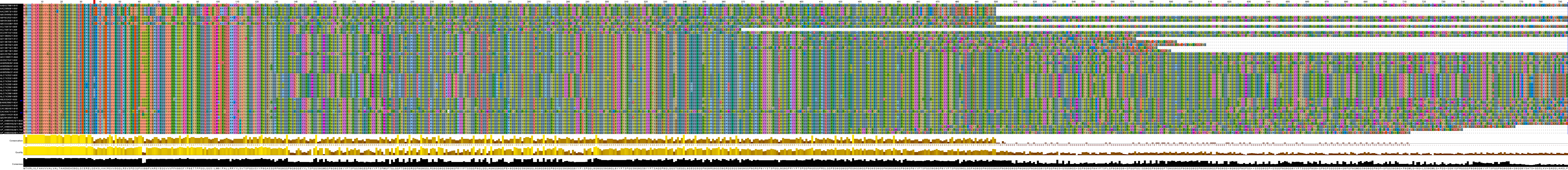

Supplement: Supplementary file 1 [file plants-12-01416-s001.zip › Supplementary Figure 2.png]

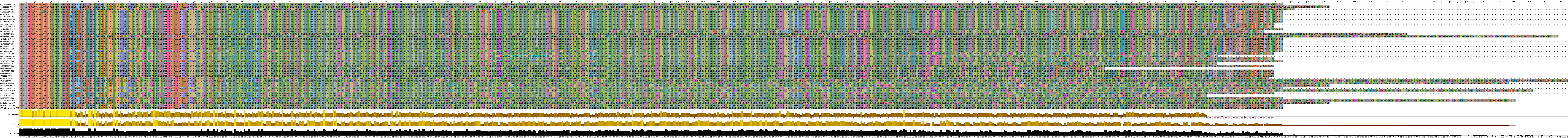

Supplement: Supplementary file 1 [file plants-12-01416-s001.zip › Supplementary Figure 3.png]

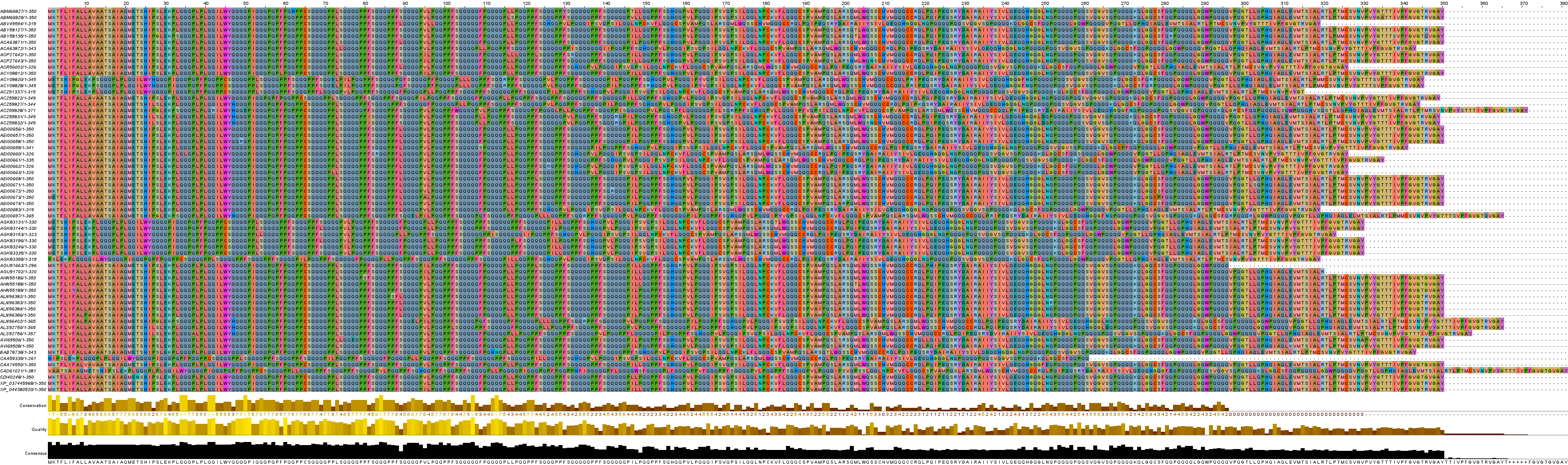

Supplement: Supplementary file 1 [file plants-12-01416-s001.zip › Supplementary Figure 4.png]
